# Contributions of Lower Structures to Higher Cognition: Towards a Dynamic Network Model

**DOI:** 10.3390/jintelligence11060121

**Published:** 2023-06-14

**Authors:** William Saban, Shai Gabay

**Affiliations:** 1 Center for Accessible Neuropsychology, Sagol School of Neuroscience, Tel Aviv University, Tel Aviv 69978, Israel; 2 Department of Occupational Therapy, Sackler Faculty of Medicine, Tel Aviv University, Tel Aviv 69978, Israel; 3 Department of Psychology, the Institute of Information Processing and Decision Making, University of Haifa, Haifa 3498838, Israel; sgabay@psy.haifa.ac.il

**Keywords:** subcortex, higher cognition, numerical cognition, attention, SEED hypothesis, evolution, dynamic network

## Abstract

Researchers often attribute higher cognition to the enlargement of cortical regions throughout evolution, reflecting the belief that humans sit at the top of the cognitive pyramid. Implicitly, this approach assumes that the subcortex is of secondary importance for higher-order cognition. While it is now recognized that subcortical regions can be involved in various cognitive domains, it remains unclear how they contribute to computations essential for higher-level cognitive processes such as endogenous attention and numerical cognition. Herein, we identify three models of subcortical–cortical relations in these cognitive processes: (i) subcortical regions are not involved in higher cognition; (ii) subcortical computations support elemental forms of higher cognition mainly in species without a developed cortex; and (iii) higher cognition depends on a whole-brain dynamic network, requiring integrated cortical and subcortical computations. Based on evolutionary theories and recent data, we propose the *SEED hypothesis*: the Subcortex is Essential for the Early Development of higher cognition. According to the five principles of the *SEED hypothesis*, subcortical computations are essential for the emergence of cognitive abilities that enable organisms to adapt to an ever-changing environment. We examine the implications of the *SEED hypothesis* from a multidisciplinary perspective to understand how the subcortex contributes to various forms of higher cognition.

## 1. Introduction

Cognition is a broad term, used to refer to the montage of mental processes that organisms use in variable environments. Although a classic cognitive psychology textbook is likely to cover a broad range of our mental capacities, we generally think of the terms in a hierarchical manner, treating the cognitive capacity required of an ant to find its way back to the nest as “simpler” than that required of the mountaineer planning a three-week trek in the Himalayas. Reflecting our species-centric belief that humans sit at the top of the cognitive pyramid, we point to various metrics of brain anatomy (e.g., size or surface area) as correlates of intelligence. Notably, with a few exceptions (Ullman 2001; Conway 2020; Walenski et al. 2007), most models of complex cognition neglect subcortical circuits while focusing mainly on the cortex.

Implicit by “neglect” is the assumption that the subcortex is of secondary importance when considering higher-level cognition, such as attention and math abilities. While research from the past decade has provided evidence that subcortical regions are engaged in some cognitive tasks (Janacsek et al. 2022; Saban et al. 2018c, 2021a), it is still a question of how (vs. if) the subcortex contributes to the computations essential for higher cognitive processes, with a focus on endogenous attention and numerical cognition.

In this paper, we suggest that the neuroscientific literature still needs to: (i) expand our explorations regarding the functional contributions of subcortical regions to higher cognition; (ii) define, explicitly, the role of subcortical mechanisms in higher cognition; and (iii) portray models that will map how the brain, as a whole, supports higher cognition. Taking an evolutionary perspective and recent empirical data, we suggest an integrative model for the functional role of subcortical regions in cognition, which highlights the evolutionary development of cortical–subcortical relations.

Across domains of study, the development of the cortex has been seen as fundamental for the emergence of the complex cognitive operations required for tasks such as volitional attention and arithmetic (Arsalidou and Taylor 2011; Corbetta et al. 2000; Frith and Dolan 1996). For instance, from this traditional perspective, a hallmark of human cognition is our ability to perform complex arithmetic operations that are mainly dependent on frontoparietal cortical regions (Arsalidou and Taylor 2011; Dehaene et al. 2004). As an historical example, neuropsychological observations from the 19th century led to the classic “Broca–Wernicke–Lichtheim–Geschwind” model of language (Geschwind 1970; Poeppel 2014), which focuses mainly on cortical layers of the brain.

The more advanced cognitive abilities of primates have been explained by the relatively larger expansion of the neocortex (Jerison 1985; Van Horik et al. 2012), and specifically the association cortex, with the apex manifest in the human brain (Bush and Allman 2004; Parks and Smaers 2018). From this phylogenetic perspective, it is appealing to adopt the view that the behavioral and cognitive differences between humans and other species can be attributed to our exceptionally large neocortical tissue.

Brodmann estimated that the prefrontal cortex represents a larger percentage of the cortex in humans (29%) than in the chimpanzee (17%) and the rhesus monkey (11.5%) (Brodmann 1902). From an ontogenetic perspective, the most complex cognitive capabilities of humans are associated with brain regions that show relatively protracted maturation, such as frontal cortical regions. For example, significant changes in white and gray matter in frontoparietal regions continue into late adolescence (Colver and Longwell 2013; Lebel and Beaulieu 2011) and, presumably, support the emergence of higher cognition.

While there is compelling evidence supporting the essential role of the neocortex in sophisticated cognition, to date, there has been little consideration of the role of subcortical structures in higher-order cognition (Janacsek et al. 2022; Parvizi 2009; but see (Ullman 2001; Conway 2020)). Subcortical regions, which are defined as the gray matter structures below the cortex, include areas such as the cerebellum, thalamus, and basal ganglia (BG). The term “higher-order cognition” refers to complex abilities such as endogenous attention, language, and math. 

## 2. The Cortico-Centric Bias

In a review of this topic, Parvizi (Parvizi 2009) suggested that neuroscience and neurology are “cortico-centric”—a conceptual bias that minimizes the possibility that “higher” functions might also depend on “lower” structures. Parvizi wrote, “*We do not have sufficient knowledge about the mode of subcortical involvement in cognition and behavioral regulation. In fact, we know very little about the role of subcortical structures in these ‘higher’ functions, precisely because a significant proportion of current research does not see beyond the cerebral neocortex*”. Parvizi also notes that some key neuroscientific methods are biased to highlight cortical mechanisms. The use of electroencephalography, magnetoencephalography, near-infrared spectroscopy, optical imaging, and transcranial magnetic stimulation (TMS) has typically been limited to the cortex. Whereas scalp-recorded electroencephalography (EEG) is considered to be cortico-centric, other electrophysiological methods such as intracranial EEG and single unit recording can provide valuable information about both cortical and subcortical activity. Furthermore, studies have shown that TMS of the cerebellum is feasible (Fernandez et al. 2020). In addition to Parvizi’s claims regarding methodological bias, one of the most commonly used methods for exploring the neural substrates of cognition also suffers from a “cortico-centric” tendency. A fundamental methodological limitation of functional magnetic resonance imaging (fMRI) is its poor ability to detect activations in subcortical structures (LaBar et al. 2001), mainly because the structures are small and susceptible to artifacts. 

Moreover, there is a self-fulfilling prophecy at play in this literature: Given assumptions that higher-order cognition is associated with cortical mechanisms, scanning protocols are frequently chosen in a way that results in minimal or suboptimal coverage of the subcortex, especially the cerebellum. Thus, the results from this work are biased and prone to overemphasizing the neocortex’s role in cognitive processes at the expense of the subcortex.

**Figure 1:**
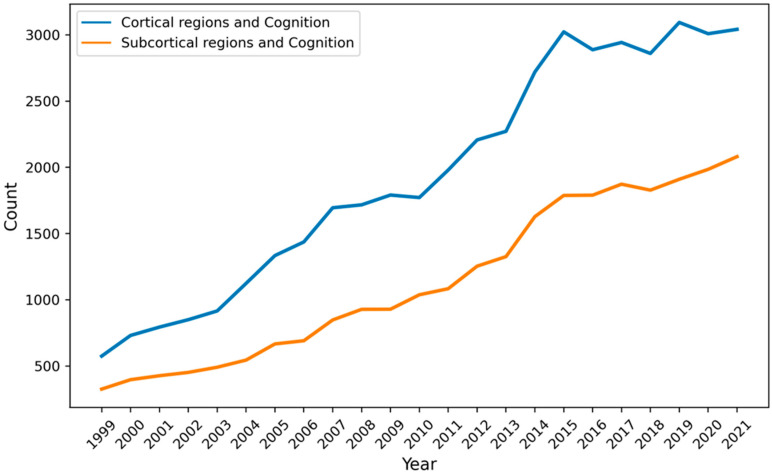
The number of PubMed results as a function of year when using the keywords “cognition” and cortical regions (frontal, parietal, temporal, and occipital lobe) compared with “cognition” and subcortical regions (cerebellum, basal ganglia, superior colliculus, and thalamus).

More than a decade after Parvizi’s provocative review, our knowledge of how subcortical regions contribute to high-order cognition remains quite murky (see also Janacsek et al. 2022). The “cortico-centric myopia” (Parvizi 2009) still appears in the current literature. Indeed, over the last two decades, there are consistently more PubMed results on cognition and cortical regions than cognition and subcortical regions (see Figure 1).

## 3. Undermining the Cortex’s Exclusive Role in Cognition

We propose to develop a granular understanding of how subcortical structures contribute to higher-level cognitive processes with a focus on endogenous attention and numerical cognition. Empirical investigations focusing on the role of subcortical regions in high-level cognitive processes are essential, not only to clarify the functional domain of different subcortical structures but also to provide a more integrative view of the cortico-subcortical networks that support cognition. In addition to the reasons described above, there are several rationales to undermine the exclusive role of the cortex in cognition. 

One critical method to explore how subcortical regions contribute to cognition is studying non-human species, which do not have fully developed cortical regions. However, the evolutionary milieu shaping cognition varies greatly across species, making it difficult to design experiments that assay cognition in a way that allows for easy comparisons between human and non-human cognition. Most animals cannot perform the typical tasks employed to study human cognition, or at least require idiosyncratic methodologies. For example, tasks designed to study higher-order cognition typically rely on explicit instructions, an option that is not possible in studies with other species. More importantly, while there are undoubtedly species-general cognitive abilities (e.g., attention, memory) that can provide a point of comparison between species, there are many species-specific abilities (e.g., echolocation in bats, haptic sensing in spiders) that preclude between-species comparisons. In addition, an organism’s cognition is formed within their particular species-specific environment. This environment may restrict, to some level, the development of their cognitive repertoire. For instance, why should the house cockroach learn to ride a bicycle, as humans do? It is of no relevance to them in their specific environment. Thus, measuring animals’ cognitive abilities requires consideration of their specific ecology. The variance in cognition across species requires an extensive set of tools and perspectives that go well beyond gross neuroscientific measures such as brain size (Van Horik et al. 2012).

It is reasonable to assume that as species evolved, a wide range of cognitive processes were necessary for survival in an ever-changing environment. Indeed, organisms that do not possess a developed cortex (e.g., fish, honeybees, spiders) are capable of what we consider high-level cognition, such as attention, executive functions, and numerical abilities (Agrillo et al. 2012; Clayton and Emery 2015; Güntürkün and Bugnyar 2016; Saban et al. 2017). Whether through adaptations arising from a common ancestor (homology) or adaptations reflective of convergent evolution, different species have developed critical cognitive abilities to survive even without a developed neocortex.

Even the traditional assumption that qualitative advances in cognition accompany an increase in brain size can be questioned (Roth and Dicke 2005). Studies involving bats, goats, and fish indicate that some mammalian brains have undergone dramatic evolutionary reductions in size (Niven 2005). Rather than an increase in brain size, interconnectivity may be a more prominent feature for explaining cognitive development (Chittka and Niven 2009). While functional connectivity studies have become quite prominent in studies of human cognition, the focus has remained on cortico–cortico connectivity. Expanding this line of work to consider cortical–subcortical networks is likely to be fruitful for a more comprehensive view of complex cognition. 

The “traditional” perspective of cognitive neuroscience is based on the premise that subcortical regions have no role in higher cognition such as attention, language, and mathematics (Arsalidou and Taylor 2011; Bush and Allman 2004; Corbetta et al. 2000; Dehaene et al. 2004; Geschwind 1970; Poeppel 2014). Researchers of subcortical structures have often focused on non-cognitive rather than cognitive functions (Koziol and Budding 2009; Noback et al. 2005). This bias for non-cognitive functions, which has historical roots (e.g., “the reptilian brain”; MacLean 1988), is also prevalent in the neuropsychology community; for example, clinicians tend to highlight motor rather than cognitive impairments in BG and cerebellar disorders (Jankovic 2008; Whaley et al. 2011; see also Janacsek et al. 2022).

## 4. Where Does the Subcortex Fit in a Model of Cognition? 

In this section, we consider different perspectives concerning the role of the subcortex in cognition. We identified three main models in the current literature (see Table 1). The first, which we consider a “traditional” perspective, is based on the premise that subcortical regions have no role in higher cognition. The second advocates a role for the subcortex in some elemental forms of higher cognition, but mainly in species that do not possess a developed neocortex. The third model proposes that higher cognition depends on a dynamic, whole-brain network that requires the integrated computations of the cortex and subcortex. Although we assume these models are relevant for a wide range of cognitive domains, we will focus on two domains, attention and numerical cognition, in evaluating the viability of these three models. 

With respect to attention, a basic dichotomy has been made between exogenous (lower-order) and endogenous (higher-order) orienting of attention (Klein and Lawrence 2011). In orienting attention tasks, participants are presented with a cue followed by a peripheral target to which they are instructed to respond. When studying exogenous orienting, the peripheral location where a target might appear is stimulated by a cue that is uninformative about the location of the upcoming target. Exogenous attention is assumed to be driven by bottom-up stimulation and is a fast and reflexive process. However, when studying endogenous attention, a property of a central cue (e.g., the cue’s color), which does not stimulate the possible target locations, provides information about the target’s location. This information of the cue needs to be learned and has a symbolic internal value. The information is aligned with the participant’s goal and is internally selected for further processing. Endogenous, or top-down, attention has been considered to be a voluntary process. Whereas exogenous attention has been linked to both subcortical and cortical regions, endogenous attention has been considered to require computations performed by the neocortex (Corbetta et al. 2000; Peelen et al. 2004). With respect to numerical cognition, the literature has long emphasized the role of neocortical regions, both for non-symbolic (e.g., quantity discrimination) and symbolic (e.g., arithmetic) numerical abilities (Arsalidou and Taylor 2011; Arsalidou et al. 2018). 

By the first model, or what we will call the *Solitary Cortex Model*, higher cognition is exclusively associated with cortical processing. The absence of the subcortex in this literature may reflect a belief that the subcortical operations are irrelevant for cognition or benign neglect, a failure to consider the subcortex in the development of neurofunctional models. This model predicts the following: (i) Species that do not have developed cortical regions (e.g., honeybees, fish) will not exhibit higher cognitive abilities since their brain lacks the anatomical substrate that enables these abilities. (ii) The human subcortex is not functionally involved in higher cognitive abilities. Based on this hierarchical perspective, neocortical regions exclusively allow the emergence of higher cognitive abilities, with subcortical regions only channeling information to the cortex for more complex computations. For example, studies of attention have highlighted how subcortical visual areas (e.g., superior colliculus (SC)) play a role in reflexive, exogenous attention, whereas volitional, endogenous attention is controlled solely by frontoparietal cortical regions (Corbetta et al. 2000; Peelen et al. 2004). Similarly, for arithmetic, a frontoparietal network is assumed to be essential for numerical abilities such as discriminating between quantities, estimating ratios, ordering numerals, and arithmetic operations (Grabner et al. 2009; Dehaene and Cohen 1997; Dehaene et al. 2003, 2004; Andres et al. 2011; Ansari and Dhital 2006; Arsalidou and Taylor 2011). This neurofunctional literature has not considered the possible role of subcortical regions in symbolic abilities, such as endogenous attention and arithmetic.

The second model is the *Cortical Superiority Model*. While variants of this model acknowledge the potential role of the subcortex, this literature emphasizes the dominance of the cortex by suggesting the following: (i) For species that lack developed cortical regions, functional computations supporting cognition can occur in subcortical substrates. (ii) For species with a developed cortex, higher cognitive abilities depend mainly on neocortical activity, supplanting more ancient subcortical computations. According to this model, the evolution of the cortex allowed new regions to take over potential contributions of subcortical regions to cognition. The subcortex may be important to support elementary processes such as processing sensory input, orienting to salient stimuli, and responding in a reflexive manner. Thus, the cortex-less housefly can discriminate between an appetizing piece of fruit and a threatening fly swatter to trigger the appropriate reflexive response (consume or flee). A species with a cortex can build upon this limited behavioral repertoire, suppressing these elementary subcortically controlled responses to produce more adapted behaviors. For example, cortical regions can integrate the current sensory input with the organism’s internal states, desires, and goals (see Finger 1994). An ontological variant of this model might be that human infants are born with a “bundle of reflexes” that, through development, come under cortical inhibitory suppression that enables goal-directed behavior (Rafal and Henik 1994; delay of gratification, for a review see Mischel et al. 1989). 

In contrast to the *Solitary Cortex Model*, the *Cortical Superiority Model* suggests that subcortical regions can have a functional role in higher cognitive processes, but this will be most evident in species that lack cortical tissue. The *Cortical Superiority Model* can account for evidence showing the essential role of the subcortex in species without a developed cortex (Güntürkün and Bugnyar 2016) in endogenous attention (Saban et al. 2017) and numerical abilities (Gross et al. 2009; Agrillo et al. 2010). This model also accounts for evidence showing only cortical activity for such operations in species with a cortex (Westerberg and Klingberg 2007; Frith and Dolan 1996; Dehaene et al. 2004; Corbetta et al. 2000). 

The third model, the *Dynamic Network Model*, emphasizes that complex cognitive abilities are a product of an interactive network that spans the cortex and subcortex. By this model, subcortical regions have a functional role in higher cognition, with the following predictions: (i) Species that do not have a developed cortex will be capable of certain higher cognitive processes, those that can be supported by the subcortex. (ii) In species with a developed cortex, subcortical structures will continue to perform their specialized computations, and through their interactions with the cortex, these computations can enable more elaborated, and in some cases, qualitatively distinct cognitive operations. (iii) Computations that are supported by the subcortex will be found in similar form in many species. 

The *Dynamic Network Model* offers an interactive perspective on endogenous attention. By this view, subcortical computations will support aspects of endogenous orienting across species, independent of how developed their cortical layers are. Behavioral studies, using the classic Posner task, have demonstrated that endogenous orienting of attention depends on subcortical visual regions (e.g., SC or thalamus) both in humans (Saban et al. 2018a, 2018b) and archer fish (Saban et al. 2017; You and Mysore 2020). However, in humans, the engagement of endogenous attention can last for longer durations than in fish (Saban et al. 2017), probably because of additional cortically based computations (Peelen et al. 2004) to maintain goals. 

In the field of numerical cognition, humans can rapidly enumerate and add collections of objects, as well as compare the numerosity of different sets. Collins et al. (Collins et al. 2017) used a stereoscopic procedure to identify visual computations that can be performed subcortically. With this method, they showed that monocular pathways in the subcortex are sufficient to support quantity discrimination. Interestingly, the contribution of subcortical regions (e.g., SC) was only evident when the judgments were made on non-symbolic stimuli (e.g., clusters of dots), but not when the quantities were specified with Arabic numbers. Consistent with these results, species that lack a developed cortex (e.g., fish, anurans, honeybees, parrots, spiders) can also make non-symbolic judgments of quantity (Agrillo et al. 2010; Gross et al. 2009; Howard et al. 2018; Nelson and Jackson 2012; Pepperberg 2006). Indeed, a recent comparative review of the neurobiology of numerical cognition concluded that numerical quantity might be processed “… *without the need to speculate on complex networks or sophisticated brains*” (Lorenzi et al. 2021), suggesting that neocortical regions are not ubiquitously essential.

Non-symbolic numerical computations may be foundational for the capacity to perform symbolic numerical computations (Dehaene 2011). For example, the operation of a non-symbolic and evolutionarily ancient system for approximation of quantity is thought to be necessary for the development of symbolic arithmetic abilities (Adams et al. 2017; Halberda et al. 2012). Thus, more complex numerical operations that are phylogenetically novel and associated with the parietal cortex (Dehaene et al. 2004) may be scaffolded from non-symbolic operations such as the Approximate Number System (ANS). The ANS, which is subcortically based (Collins et al. 2017; Lorenzi et al. 2021) and allows a general representation of numbers (Adams et al. 2017), might be necessary for the development of a counting procedure. Indeed, performance in non-symbolic approximation of quantities tasks is predictive of school success in mathematics (Starr et al. 2013). 

The *Dynamic Network Model* stands in contrast to the *Cortical Superiority Model*, in that the cortex is seen as exploiting subcortical computations, rather than suppressing them. Thus, the emphasis is on the dynamic interaction through which cortical processing has access to computations performed in subcortical regions and modulates this activity as a function of higher-level goals to support more complex, adaptive behaviors. This account predicts that with the evolution of the cortex, the functional domain of a cognitive ability can be broadened, allowing subcortical computations to be employed in a more flexible manner.

## 5. How Does the Subcortex Support the Emergence of Cognition?

In this section, we elaborate on the *Dynamic Network Model* by adding a novel conceptual framework for how the subcortex contributes to the evolution of cognition. The core hypothesis is that subcortical computations are essential for the development of a new cognitive function. We suggest a general neural hypothesis by which the Subcortex is Essential for the Early Development (SEED) of complex cognition. 

According to our *SEED hypothesis*, since subcortical regions developed early in evolution and have remained functional up to the present, they are essential for the emergence of cognitive computations that enable organisms to adapt to an ever-changing environment. The *SEED hypothesis* has five principles.

First, new cognitive skills do not emerge in isolation; rather, each new cognitive function builds upon previous cognitive computations. Second, subcortical computations serve as the scaffolding for developing new cognitive functions. Third, similarly to other theories (Janacsek et al. 2022), a subcortical structure can support many functions via core computations. Fourth, a given function can be supported by many structures. Fifth, cortical regions have access to subcortical computations and can exploit them to establish a dynamic neural network, which enables more adaptive cognitive representations.

The *SEED hypothesis* is aligned with previous theories and empirical evidence. There is converging evidence that subcortical structures, such as the BG and cerebellum, play an essential role in the proceduralization of both motor and cognitive processes (Knowlton et al. 2016; Frank et al. 2004; Pascual-Leone et al. 1993; Callu et al. 2013; Molinari et al. 1997). Different subcortical structures are involved in the learning of skills through repeated practice and reinforcement over time (Knowlton et al. 2016; Nicolson et al. 2010; Pascual-Leone et al. 1993; Saban et al. 2017, 2018b). Subcortical and cortical structures act as a feedback loop (Bostan and Strick 2018) to facilitate processes, enabling efficient and automatic execution of tasks. Both in humans and other species, subcortical computations can be repurposed and manipulated, allowing cortical regions access to previous computations. We propose that a subcortical region serves as an essential evolutionary “*seed*”, allowing proceduralization of processes required for the emergence of a new cognitive function. Therefore, subcortical computations can allow the development of new expertise in various domains, from orienting of attention to playing the piano or solving math problems.

The *SEED hypothesis* emphasizes a neural principle of reuse and accessibility, which provides a useful framework for considering dynamic interactions between the subcortex and cortex. In order to develop new cognitive abilities, the brain has to find a “*neuronal niche*”, a set of circuits that are sufficiently close to the required function (Dehaene and Cohen 2007). These niches frequently come from ancient subcortical structures. Existing subcortical neural mechanisms can exert a powerful influence on the development of cognitive function. We propose that subcortical computations for one function can be accessed and reused to form a novel skill.

Several theories are in line with this principle of neural reuse and accessibility. Paul Rozin (Rozin 1976) proposed that computations that initially evolved to solve specific problems become accessible to other systems through evolution as well as within the individual lifetime of an organism. Change or expansion of a function, because it is more generally available or accessible, “*would have adaptive value when an area of behavioral function could profit from programs initially developed for another purpose*”. This idea has been reframed and elaborated upon in Gallese’s “neural exploitation” hypothesis (Gallese and Cuccio 2018), Hurley’s “shared circuits model” (Hurley 2008), Dehaene’s “neuronal recycling” theory (Dehaene and Cohen 2007), and Anderson’s “massive redeployment” hypothesis (Anderson 2007). The core idea is that neural networks are dynamic and acquire new uses after establishing an initial function.

For instance, subcortical computations to support reflexive orienting of attention (exogenous) can be used to develop an endogenous system, one that can operate on symbolic inputs (Carrasco 2011; Klein and Lawrence 2011). Similarly, subcortical non-symbolic numerical computations of quantity can be exploited for the emergence of symbolic arithmetic procedures such as counting (Rugani et al. 2009; Lorenzi et al. 2021).

The hypothesis that complex cognition emerges from ancient, elementary operations has been discussed in the literature examining the relationship between tool use and language (Corballis 1991; Thibault et al. 2021). Corballis (Corballis 1991) proposed that our unique ability for language was scaffolded from a system that had the computational capacity to build extended and flexible representations, or what is called a “*generative learning device*” (GAD). The GAD enables us to generate forms and meanings from a few elements, providing the basis for both language and tool use skills. While Corballis proposed that the GAD reflected a specialization of the left hemisphere, a recent fMRI study has shown that the syntactic operations associated with tool use and language activate the BG, one major subcortical structure (Thibault et al. 2021). Interestingly, learning a novel tool use task was also found to improve performance in a complex language task, pointing to shared computations across these two domains.

Similarly, in a recent study we demonstrated that subcortical mechanisms—lower visual channels—play a causal role in cognitive transfer of complex skills such as symbolic arithmetic (Saban et al. 2021b). We found that exposing only one monocular channel to a visuospatial training resulted in a larger transfer effect in the trained monocular channel compared with the untrained monocular channel. Such cognitive transfer was found between spatial abilities and mathematical subtraction problems (far transfer). Importantly, the benefits of the trained eye were not observed in old problems nor in other tasks that did not involve visuospatial abilities (the Stroop task, a multiplication task). These results challenge the exclusive role of the cortex in cognitive transfer and complex arithmetic. 

These recent studies not only further support the evolutionary relation between different cognitive abilities (tool use and language or visuospatial and math), but also strengthen the notion that the subcortex mediates the development of complex cognition (i.e., language and math). In addition, the results suggest a new subcortical mechanism for the emergence of cognitive skills, which could be shared across different species.

Whereas the *SEED hypothesis* focuses on how new cognitive abilities emerge over the course of evolution, it is important to recognize that ancient subcortical computations continue to be essential for higher cognition, the critical idea of the Dynamic Network Model. Indeed, researchers have found that the subcortex contributes to many higher cognitive functions in humans spanning attention, arithmetic, language, executive functions, and more (Collins et al. 2017; Conway 2020; Gabay and Behrmann 2014; Saban et al. 2017, 2018, 2019, 2021a, 2021b; Soloveichick et al. 2021; Ullman 2001; Walenski et al. 2007). In addition, higher cognitive functions are exhibited by many different organisms, including species that do not possess developed cortical structures, such as insects, pigeons, and fish (Agrillo et al. 2010; Gross et al. 2009; Güntürkün and Bugnyar 2016; Howard et al. 2018; Kattner et al. 2017; Portavella et al. 2004; Saban et al. 2017; Schlegel and Schuster 2008). 

Similarly, subcortical regions play a role in complex cognitive abilities during childhood when the cortex is still developing. For instance, the BG and cerebellum are involved in language and numerical abilities in studies examining developmental language disorder, dyslexia, and dyscalculia (Evans and Ullman 2016; Leisman et al. 2014; Lum et al. 2013; Nicolson et al. 2010; Nicolson and Fawcett 2007; Nicolson et al. 2001; Ullman et al. 2020). It has been suggested that a number of developmental disorders (e.g., DLD, dyslexia) might be partly explained by a procedural circuit deficit (Ullman et al. 2020), including the BG. Together, these studies further support our notion that subcortical regions have a role in the proceduralization of processes (see also Ullman et al. 2020; Evans and Ullman 2016). Thus, impairment in a subcortical structure can lead to a procedural deficit.

**Figure 2:**
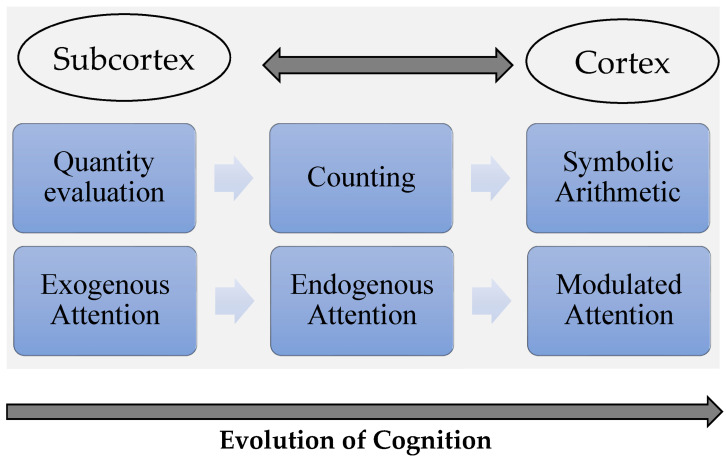
Foundational computations supported by the subcortex (e.g., quantity evaluation, exogenous attention) can be repurposed to develop new cognitive abilities (counting, endogenous attention). The cortex has access to subcortical computations, creating a dynamic network that allows for the emergence of complex cognition (symbolic arithmetic, modulated attention). Future studies should examine the potential contributions of subcortical structures to other cognitive computations (e.g., carrying) and how they are related to more basic functions (e.g., working memory).

Figure 2 below depicts how the *SEED hypothesis* explains the development of new cognitive functions over the course of evolution. There are bidirectional relations between the cortex and the subcortex. The subcortex is essential for the early development of higher cognitive abilities, which depend on previous essential cognitive computations. The role of the subcortex is to serve as a scaffolding needed for the emerging cognition. The cortex accesses different subcortical computations and can orchestrate a wider range of operations. 

## 6. Methods for Subcortical–Cortical Investigations

In order to explore the contribution of the subcortex to complex cognition, researchers will need to utilize a range of methods (see Table 2). First, behavioral methods can dissociate the contributions of subcortical and cortical processing to task performance. Visual information is monocularly segregated until it reaches binocular neurons in extrastriate regions of the visual cortex (Menon et al. 1997). Hence, visual input from the two eyes is segregated in the subcortex. A stereoscope can be used to create dichoptic stimuli in which each eye receives distinct information. By comparing performance between conditions solely dependent on monocular processing channels and conditions in which the information is integrated between the two eyes, one can make inferences about the kinds of visual computations that can be performed subcortically. This approach has been used to demonstrate the *causal* role of visual subcortical channels in attention, arithmetic, and other cognitive processes (Collins et al. 2017; Gabay and Behrmann 2014; Saban et al. 2018a, 2019, 2021a; Soloveichick et al. 2021). 

Second, perturbation methods such as lesion studies provide a means to evaluate the causal role of a specific brain region in a cognitive function. To date, much of this work has been descriptive, designed to ask if damage to a particular region impairs performance in a particular cognitive domain. To move to the more nuanced question of *how* these regions contribute to task performance, these neuropsychological investigations should be tailored to test specific functional hypotheses. The *SEED hypothesis* can provide a guide in designing such studies, seeking to isolate elementary operations that may serve as the building blocks for more complex cognition. For example, we have found that patients with cerebellar degeneration or BG dysfunction have dissociable impairments in arithmetic procedures. While neuropsychological research has traditionally involved small samples, online methods can be employed for more efficient data collection from patients with subcortical pathology (Saban and Ivry 2021; Binoy et al. 2023). 

Third, although most protocols for neuroimaging have generally been designed to maximize coverage and quality of activity in the cortex, some protocols have been developed to focus on the subcortex (e.g., Sylvester et al. 2007). Novel high-resolution scanning such as that made possible with 7T fMRI can provide a more sensitive technique to study neural activation in smaller subcortical regions (Wilkey et al. 2020). 

Finally, comparative approaches can shed light on the evolutionary development of cognition and demonstrate potential subcortical–cortical relations. Studies involving species that do not possess a developed cortex (e.g., fish) have provided important insights regarding the cognitive abilities that can be supported by the subcortex (Güntürkün and Bugnyar 2016; Leadner et al. 2020; Saban et al. 2017). Studies involving species with a more highly developed cortex (e.g., non-human primates) can shed light on the interaction of subcortical–cortical networks and how the cortex might affect subcortical cognitive computations (Dorris et al. 2002; Gao et al. 2018). Finally, comparing neural connectivity between species and correlating it with different measures of cognitive abilities offers a way to test the dynamic network model. 

## 7. Conclusions

More than a decade ago, Parvizi (2009) raised concerns with a “cortico-centric” view of the human brain, advocating for a broader perspective that recognized the vital contributions of the subcortex to cognition. One such approach to the organizational principle of the brain centers on the idea of neural reuse, whereby computational specializations that evolved to solve a specific problem become exploited in a different task domain (Anderson 2010; Rozin 1976). We believe that our SEED hypothesis provides a parsimonious and fruitful framework for considering how the subcortex and cortex interact to enable complex cognition. From an evolutionary perspective, we view the subcortex as providing the “seeds” for higher-order cognition, being used to build more elaborate operations. Understanding higher cognition requires consideration of whole-brain dynamics. Our perspective advocates for a theoretical shift from a classical perspective of the neocortex as the hub of high-level cognition to a more integrated view in which our cognitive abilities reflect the interplay between subcortical and cortical neural mechanisms.

## Figures and Tables

**Table 1:** Summary of the three models.

	Model	Solitary Cortex	Cortical Superiority	Dynamic Network
Feature				
Subcortical involvement in humans		NO	NO	YES
2. Subcortical involvement in species without a developed neocortex		NO	YES	YES
3. Neocortical regions’ influence on subcortical regions		NO influence	Superiority (suppression/migration)	Dynamic (modulation/joint work)

**Table 2:** Four methods for subcortical investigations.

Method	The Main Benefits of the Method	Question Addressed
(1) Dichoptic presentation	It enables us to examine the functional contribution of monocular neural substrates (mostly subcortical) in human cognition.	What is the involvement of monocular regions in a cognitive function?
(2) Perturbation methods such as lesion studies	They allow us to infer the specific causal contribution of a given subcortical mechanism.	What is the role of a specific subcortical region in a cognitive function?
(3) Imaging studies focusing on subcortical regions	They allow us to detect neural activity of subcortical regions and the neural dynamics of subcortical–cortical networks.	What is the role of different subcortical regions in a cognitive function and the interactions between cortical and subcortical regions?
(4) Animal cognition studies	They provide insights regarding the cognitive ability of a neural system lacking a developed cortex.	What is the evolutionary origin of a cognitive function?

## Data Availability

Not applicable.
